# Food insecurity and youth suicidal behaviours: Evidence from the Canadian Health Survey of Children and Youth

**DOI:** 10.17269/s41997-025-00998-7

**Published:** 2025-02-24

**Authors:** Lilia Lounis, Lovena Jacqdom, Frank J. Elgar

**Affiliations:** 1 Department of Psychology, McGill University, Montreal, Quebec Canada; 2 Department of Psychiatry, McGill University, Montreal, Quebec Canada; 3 Department of Equity, Ethics and Policy, McGill University, Montreal, Quebec Canada

**Keywords:** Food insecurity, Mental health, Suicide, Adolescent, Poverty, Insécurité alimentaire, Santé mentale, Suicide, Adolescent, Pauvreté

## Abstract

**Objective:**

Youth suicide, a major cause of death, is linked to poverty and other adverse experiences. Evidence of its association with food insecurity is unclear due to inadequate controls for household income in previous research. This cross-sectional study used independent reports of household income, food insecurity, and suicidal behaviours to examine these associations in a population-based sample.

**Methods:**

The 2019 Canadian Health Survey on Children and Youth surveyed 6735 youth (15–17 years), gathering data on sadness/hopelessness, suicidal ideation, and suicide attempts. Adults provided information on household food insecurity using a multi-item scale and about diagnosed mood disorders in youth. Household income data were provided by government tax records. We used Poisson regressions to estimate the relative risk (RR) of each suicidal behaviour attributed to household food insecurity, adjusting for household income and other covariates.

**Results:**

Approximately one in five (19.8%) youth experienced marginal (5.2%), moderate (7.9%), or severe (3.3%) food insecurity. Moderate or severe food insecurity was associated with increased risks of sadness/hopelessness, mood disorder, suicide ideation, and suicide attempts (RRs 1.30–2.17) after controlling for household income differences and other covariates. Generally, more severe food insecurity was positively associated with suicide behaviours.

**Conclusion:**

Household food insecurity is associated with youth suicidal behaviours, independently of household income. Its underlying pathway to youth mental health includes social and psychological factors that require targeted policy intervention.

**Supplementary Information:**

The online version contains supplementary material available at 10.17269/s41997-025-00998-7.

## Résumé

**Objectif:**

Le suicide chez les jeunes, l’une des principales causes de mortalité, est associé à la pauvreté et à d’autres expériences défavorables. Cependant, le lien entre l’insécurité alimentaire et les comportements suicidaires demeure insuffisamment documenté, notamment en raison d’un contrôle inadéquat des revenus des ménages dans les recherches précédentes. Cette étude transversale explore ces associations en utilisant des données indépendantes sur les revenus des ménages, l’insécurité alimentaire et les comportements suicidaires, dans un échantillon représentatif de la population.

**Méthodes:**

L’Enquête canadienne sur la santé des enfants et des jeunes de 2019 a interrogé 6735 jeunes (15–17 ans), recueillant des données sur la tristesse/le désespoir, les pensées suicidaires et les tentatives de suicide. Les adultes ont fourni des informations sur l’insécurité alimentaire des ménages à l’aide d’une échelle multi-items et sur les troubles de l’humeur diagnostiqués chez les jeunes. Les données sur le revenu des ménages ont été obtenues à partir des dossiers fiscaux du gouvernement. Des régressions de Poisson ont été utilisées pour estimer les risques relatifs (RR) associés à chaque comportement suicidaire attribué à l’insécurité alimentaire, en tenant compte des différences de revenus des ménages et d’autres covariables.

**Résultats:**

Près d’un jeune sur cinq (19,8 %) a été exposé à une situation d’insécurité alimentaire marginale (5,2 %), modérée (7,9 %) ou sévère (3,3 %). L’insécurité alimentaire modérée ou sévère est associée à une augmentation des risques de tristesse/désespoir, de troubles de l’humeur, d’idées suicidaires et de tentatives de suicide (RR compris entre 1,30 et 2,17), après ajustement pour les différences de revenus des ménages et d’autres covariables. En général, une relation positive entre la sévérité de l’insécurité alimentaire et les comportements suicidaires a également été observée.

**Conclusion:**

L’insécurité alimentaire des ménages est associée aux comportements suicidaires chez les jeunes, indépendamment des revenus des ménages. Ces résultats suggèrent des mécanismes sous-jacents, incluant des facteurs sociaux et psychologiques, qui nécessitent des interventions politiques ciblées.

## Introduction

Suicide is the fourth leading cause of death in youth and adults (Bertuccio et al., 2024; World Health Organization, 2024). Suicidal behaviours, including suicidal thoughts and planning, typically first emerge in adolescence and are more prevalent in girls than in boys (Bertuccio et al., 2024; Nock et al., 2008). Their presence increases the risk of subsequent attempts and death (Georgiades et al., 2019). About one third of youth who have suicidal thoughts attempt suicide within a year (Turecki & Brent, 2016). Following hospitalization after a suicide attempt, the risk of subsequent suicide death is 1.6% in the next year and 3.9% over the next 5 years (Carroll et al., 2014). Previous research has identified several precipitating factors to suicide that include social isolation, binge drinking, drug use, mental illness, abuse, and violence (Bilsen, 2018; Georgiades et al., 2019; Turecki & Brent, 2016). The strongest predictors of a suicide attempt are self-harm, suicidal thoughts, and feelings of hopelessness (Victor & Klonsky, 2014). However, not all risk factors differentiate individuals who think about suicide from those who plan and attempt, which is why it is important to understand the social determinants of each of these behaviours (Georgiades et al., 2019; Victor & Klonsky, 2014).

Epidemiological research on social patterns in youth suicidal behaviours supports prevention efforts. An emerging line of research in this area investigates the association of food insecurity and mental health. Having insufficient access to safe and nutritious food is a problem that affects over 2 billion people globally (Food and Agriculture Organization, 2023). In the Canadian Community Health Survey (CCHS), about one in every seven Canadian youth was found to live with food insecurity in the household (Men et al., 2021). The rate of food insecurity in Canada has increased since the beginning of the COVID-19 pandemic, from 16% in 2021 to 18% in 2022 (Statistics Canada, 2024a). The experiences associated with food insecurity are an intense source of psychological stress for all members of a household and contribute to various mental and physical health problems (GBD 2021 Demographics Collaborators, 2024). These shared experiences may include meal skipping, concerns about securing enough food, or having to depend on food donations or waste. These are all visceral reminders of social disadvantage and can increase risks of suicidal thoughts and behaviours through social isolation, malnutrition, and chronic activation of neuroendocrine stress pathways (McLaughlin et al., 2012; Pryor et al., 2016).

A to-date overlooked question regarding youth is whether food insecurity increases the risk of suicidal behaviours after other socioeconomic factors are controlled. For instance, does experiencing food insecurity pose risks that are not fully explained by household income poverty? Although food insecurity is more prevalent in lower income households, previous research done with adult samples found that food insecurity and poverty each pose a unique risk to mental health and therefore warrant targeted intervention. In adults, food insecurity is associated with substance abuse, depressive symptoms, and suicidal thoughts after controlling for household income (Davison et al., 2015; Pryor et al., 2016). Whether these effects extend to youth is unclear. A study in Canada examined the association of household food insecurity and youth mood disorder and suicidal thoughts using data from the CCHS but did not examine previous suicide attempts (Men et al., 2021). Longitudinal studies in the United States and Canada found associations of childhood food insecurity with internalizing and externalizing problems in adolescence after controlling for persistent childhood poverty or household income insufficiency (Paquin et al., 2021; Slopen et al., 2010). Other longitudinal data in Canada show similar associations of childhood hunger with depression and suicide ideation in adolescence and early adulthood (McIntyre et al., 2013, 2017).

The Family Stress Model offers a useful framework for understanding how food insecurity can adversely affect adolescent mental health at the household level (Conger et al., 1990). According to this model, financial strain heightens parental stress, which can lead to changes in family dynamics, increased conflict between parents, and more negative parenting practices. The psychosocial consequences of a shared family stressor, in turn, negatively influence children’s emotional and behavioural development. The supporting evidence for this model shows that household food insecurity is associated with parenting stress, poor parental mental health, and poor parenting practices and these constructs mediate part of the association of food insecurity and youth emotional distress (Ashiabi & O’Neal, 2008; Kotchick et al., 2021; Marçal, 2022).

To our knowledge, no study has yet investigated the association of household food insecurity and youth-reported suicidal behaviours (including suicide attempts) with robust controls for household income. This study addressed this evidence gap using data from a national population-based survey of Canadian youth (15–17 years). The survey uniquely gathered information on household food insecurity from adults, on household income from government tax records, and on multiple suicidal behaviours from youth reports. The independence of these sources was useful for ruling out potential reporting bias in food insecurity and household income. The behaviours we examined represent key precipitating factors in suicide—sadness/hopelessness (Wolfe et al., 2019) and mood disorder (Ruch et al., 2021)—and are consistent with the ‘ideation-to-action’ framework that guides most contemporary research on suicide (Bayliss et al., 2022). This framework posits that suicide ideation and suicide attempts are related but distinct behaviours that may differ in their social determinants. We hypothesized positive associations would be found between the severity of household food insecurity and risk of each suicidal behaviour after household income differences were considered.

## Methods

### Study design and participants

The 2019 Canadian Health Survey on Children and Youth (CHSCY) is a cross-sectional household survey conducted by Statistics Canada (Statistics Canada, 2022). It recruited a stratified sample of children and adolescents (1–17 years) living in private households in all Canadian provinces and territories using the Canada Child Benefit file as the sampling frame (*n*=39,951; response rate 52.1%). The sample was stratified by province and age group and excluded youth in First Nations and other Indigenous communities, foster homes, and institutional settings. Once weighted, the data represent approximately 98% of the Canadian population aged 1 to 17 years in all provinces and 96% in all territories.

In this study, we used data on a subsample of 6735 youth (15–17 years) who were surveyed about suicidal behaviours. CHSCY survey questions on suicidal behaviour were asked only of youth in this age range. The subsample was proportionally distributed across provinces and territories. Questionnaires were completed online or by phone between February and August 2019. An adult “person most knowledgeable” (PMK) about the sampled youth provided information on sociodemographic characteristics and food insecurity. The PMKs were mostly mothers (77.7%) or fathers (20.6%) of the sampled youth (Statistics Canada, 2022).

### Variables and data sources

#### Suicide behaviours

Dichotomous (yes/no) survey items asked youth about three previous suicidal behaviours. Suicidal ideation: “These questions ask about sad feelings and attempted suicide. Sometimes people feel so depressed about the future that they may consider attempting suicide, that is, taking some action to end their own life. In the past 12 months, did you ever seriously consider attempting suicide or taking your own life?”; Suicide attempt: “Have you ever attempted suicide or tried taking your own life?”; and Sadness/hopelessness: “During the past 12 months, did you ever feel so sad or hopeless almost every day for two weeks or more in a row that you stopped doing some usual activities?”

Interviews with the PMK supplied information about diagnosed mood disorders in youth: “We are interested in long-term conditions which are expected to last or have already lasted 6 months or more and that have been diagnosed by a health professional. Has this child been diagnosed with any of the following long-term conditions: A mood disorder such as depression, bipolar disorder, mania or dysthymia? (yes/no).”

#### Food insecurity

Parent questionnaires included the Household Food Security Survey Module (HFSSM), an 18-item scale that was originally developed by US Department of Agriculture and adapted by Statistics Canada for use in the Canadian Community Health Survey (*α*=0.81; Bickel et al., 2000). Eight items describe the experiences of children in the household, ranging from “You or other adults in your household relied on only a few kinds of low-cost food to feed child(ren)” to “Any of the child(ren) ever did not eat for whole day.” Ten items describe the adults’ experiences, ranging from “You and other household members worried food would run out before you got money to buy more” to “You or other adults in your household ever did not eat for whole day.” We used Statistics Canada’s classification criteria to identify four groups (food secure and marginal, moderate, or severe food insecure; Statistics Canada, 2020). The summation of affirmative answers to the child items classified youth as either food secure (0 items) or marginally (1 item), moderately (2–4 items), or severely (5–8 items) food insecure. A summation of affirmative answers to the ten adult items classified adults in the household as either food secure (0 items) or marginally (1 item), moderately (2–5 items), or severely (6–19 items) food insecure. Household food insecurity status (marginal, moderate, or severe) was operationalized as the presence of either youth or adult food insecurity at that level.

#### Covariates

The regression analyses controlled for differences in household income (before taxes), youth age (15, 16 or 17 years), gender (boy/girl), visible minority status (white/non-white), family structure (1 or 2 parents or caregivers), and household size (1–2, 3, 4, or 5+ persons). Household income data held by the Canada Revenue Agency (T1 Family File) were linked to individual records by Statistics Canada in order to reduce respondent burden and ensure accuracy of the data (Statistics Canada, 2022, 2024b). Gender, according to the CHSCY survey, refers to “current gender which may be different from sex assigned at birth and may be different from what is indicated on legal documents” and was identified as male gender (boy), female gender (girl), or other (Statistics Canada, 2022). Statistics Canada’s vetting guidelines related to confidentiality prevented us from reporting on the group gender non-binary youth due to its small size.

### Data analysis

The data were analyzed using Stata/SE 17.0 (StataCorp, College Station, TX) at a university-based research lab (Research Data Centre) administered by Statistics Canada. Household income was log-transformed to adjust for its diminishing returns on mental health at higher values. We applied weighted Poisson regressions with robust standard errors (SEs) to estimate the relative risk (RR) and 95% confidence interval (CI) for each suicidal behaviour at marginal, moderate, and severe household food insecurity (versus household food security), with controls for the covariates (gender, age group, visible minority, household size, family structure, and household income log). While usually applied to count data, modified Poisson regressions with robust SEs are recommended for use with binary outcomes when their prevalence exceeds 10% and of interest is the amount of risk attributed to an exposure (Gnardellis et al., 2022; Zou, 2004). Odds ratios from logistic regressions are less intuitive than relative risk and found to be increasingly biased as the prevalence of the outcome increases (Knol et al., 2012).

Although missing data were few and appeared to be random, we used chained multiple imputations to estimate missing observations of age (0.1%), gender (0.7%), visible minority status (0.1%), family structure (0.2%), household income (<0.1%), food insecurity (3.5%), sadness/hopelessness (0.1%), mood disorder (0.2%), suicide ideation (0.1%), and suicide attempt (0.1%). No data were missing on household size. Ten imputed datasets were generated using iterative and chained multivariate regressions of each variable with missing data, and then the results were pooled across these datasets. Stata’s *margins* postestimation command was then used to estimate the adjusted predicted prevalence (%) of each outcome across levels of food insecurity.

## Results

Characteristics of the sample are summarized in Table 1. The sample was evenly distributed across age and gender groups. Most youth lived in households of four (39.5%) or five or more (33.3%) persons and with two caregivers (79.1%), whereas 7.1% of youths lived with one caregiver. Approximately one in five youth (19.8%) lived in food insecure households. Of these, 7.9% lived with moderate food insecurity and 3.3% lived with severe food insecurity. With respect to suicidal behaviour, an estimated 25.8% reported feelings of sadness and/or hopelessness during the past 12 months, 13.8% of youth reported suicidal ideation during the past 12 months, and 6.8% reported a previous suicide attempt (lifetime). According to PMKs, 5.6% of youths in the sample had been diagnosed with a mood disorder.

**Table 1:** Sample characteristics

	*n*	%
Age (years)		
15	2395	35.6
16	2280	33.9
17	2055	30.5
Missing	5	0.1
Gender		
Female	3265	48.5
Male	3425	50.9
Missing	45	0.7
Visible minority		
Yes	2110	31.3
No	4590	68.2
Missing	35	0.5
Household size		
2	480	7.1
3	1350	20.0
4	2660	39.5
5+	2245	33.3
Missing	0	0.0
Family structure		
Two caregivers	5325	79.1
One caregiver	1400	20.8
Missing	10	0.2
Food insecurity (FI)		
Food secure	5400	80.2
Marginal FI	350	5.2
Moderate FI	530	7.9
Severe FI	220	3.3
Missing	235	3.5
Suicidal behaviours		
Suicidal ideation (past 12 months)	930	13.8
Suicide attempt (lifetime)	455	6.8
Sadness/hopelessness	1735	25.7
Mood disorder	375	5.6

Cell counts (*n*) are rounded to the nearest 5 to conform to Statistics Canada’s disclosure rules

The regression analyses of each of these suicidal behaviours showed consistently positive associations with food insecurity (Table 2). However, the strength of these associations varied across outcomes and severity of food insecurity. For example, youth from households with marginal and moderate food insecurity were more likely to experience suicidal ideation (RR = 1.39 [95% CI 1.06, 1.81], 1.39 [95% CI 1.11, 1.73] respectively) compared to youth in food secure households. The association was not significant at severe food insecurity, possibly due to the small cell size. Lifetime suicide attempt was associated with marginal (RR = 1.46 [95% CI 1.01, 2.13]), moderate (RR = 1.65 [95% CI 1.22, 2.21]), and severe (RR = 1.65 [95% CI 1.08, 2.05]) food insecurity. Similarly, sadness/hopelessness was associated with moderate (RR = 1.37 [95% CI 1.17, 1.61]) and severe (RR = 1.30 [95% CI 1.02, 1.66]) food insecurity. Diagnosed mood disorder was also associated with food security at marginal (RR = 1.65 [95% CI 1.20, 2.28]) and severe (RR = 2.17 [95% CI 1.46, 3.22]) levels.

**Table 2:** Relative risk (RR) of suicidal behaviours and mental health problems in Canadian adolescents

	Suicide ideation (past 12 months)		Suicide attempt (lifetime)		Sadness/hopelessness		Mood disorder	
	RR	95% CI	RR	95% CI	RR	95% CI	RR	95% CI
Gender								
Female	Ref.		Ref.		Ref.		Ref.	
Male	0.55**	(0.48, 0.63)	0.48**	(0.39, 0.58)	0.51**	(0.46, 0.57)	0.36**	(0.31, 0.49)
Age								
15	Ref.		Ref.		Ref.		Ref.	
16	1.11	(0.95, 1.29)	1.05	(0.84, 1.32)	1.14*	(1.02, 1.27)	1.28	(0.99, 1.65)
17	1.09	(0.93, 1.27)	1.19	(0.95, 1.49)	1.12*	(1.00, 1.26)	1.63**	(1.28, 2.09)
Visible minority								
No								
Yes	1.03	(0.89, 1.20)	0.81	(0.64, 1.01)	1.12*	(1.01, 1.25)	0.43**	(0.32, 0.57)
Household size								
1–2	Ref.		Ref.		Ref.		Ref.	
3	0.92	(0.69, 1.22)	0.96	(0.66, 1.40)	0.96	(0.78, 1.17)	0.95	(0.65, 1.38)
4	0.83	(0.62, 1.11)	0.75	(0.51, 1.11)	0.83	(0.67, 1.02)	0.71	(0.47, 1.06)
5+	0.98	(0.73, 1.31)	0.95	(0.64, 1.42)	0.90	(0.73, 1.11)	0.62*	(0.41, 0.95)
Family structure								
Two caregivers	Ref.		Ref.		Ref.		Ref.	
One caregiver	1.25*	(1.03, 1.53)	1.34*	(1.02, 1.75)	1.23**	(1.07, 1.42)	1.12	(0.84, 1.49)
Income (log)	1.11*	(1.00, 1.24)	0.95	(0.82, 1.10)	1.03	(0.95, 1.11)	0.82*	(0.71, 0.96)
Food insecurity (FI)								
Food secure	Ref.		Ref.		Ref.		Ref.	
Marginal FI	1.39*	(1.06, 1.81)	1.46*	(1.01, 2.13)	1.18	(0.95, 1.46)	1.42	(0.93, 2.16)
Moderate FI	1.39**	(1.11, 1.73)	1.65**	(1.22, 2.21)	1.37**	(1.17, 1.61)	1.65**	(1.20, 2.28)
Severe FI	1.38	(0.99, 1.92)	1.65*	(1.08, 2.05)	1.30*	(1.02, 1.66)	2.17**	(1.46, 3.22)

*p <0.05; **p<0.01

The regressions also showed that all four outcomes were less prevalent in boys than in girls (RRs = 0.36 [95% CI 0.31, 0.49] to 0.55 [95% CI 0.48, 0.63]). The 16- and 17-year-olds (compared to 15-year-olds) showed greater risks of sadness/hopelessness (RRs = 1.14 [95% CI 1.02, 1.27] and 1.12 [95% CI 1.00, 1.26], respectively), and the 17-year-olds showed greater risk of mood disorder than the 15-year-olds (RR = 1.63 [95% CI 1.28, 2.09]). We observed no associations of visible minority status and household size, and either null or positive associations with household income (Table 2).

**Fig. 1:**
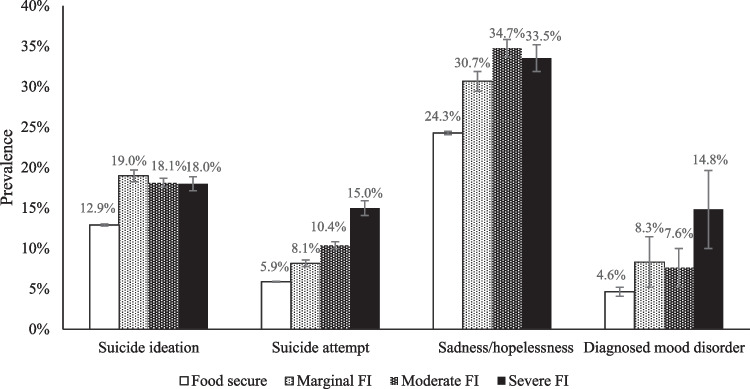
Predicted prevalence of suicidal behaviours and mental health problems by household food insecurity (FI) status in Canadian youth. Error bars indicate 95% confidence intervals

Figure 1 shows the regression-based predicted prevalence of suicidal behaviours by food insecurity status with adjustment for household income and other covariates. Food secure youth showed the lowest prevalence of any suicidal behaviour. Graded associations with the severity of food insecurity were most evident in lifetime suicide attempt (from 5.9% in food secure households to 15.0% at severe food insecurity) and diagnosed mood disorder (from 24.3% in food secure households to 33.5% at severe food insecurity).

## Discussion

This study examined contemporaneous associations of household food insecurity and youth suicidal behaviour using data from a large population-based survey. Food insecurity was associated with youth-reported suicidal ideation, suicide attempts, and feelings of sadness/hopelessness. Also, severe food insecurity was associated with the presence of a diagnosed mood disorder. These associations of food insecurity and suicidal behaviours are consistent with previous studies (e.g. Pryor et al., 2016; Slopen et al., 2010). Men et al. (2021) also found associations of household food insecurity with youth mood disorders and suicidal thoughts. We replicated this result and found a similar dose-response association between the severity of food insecurity and prevalence of suicide attempts and sadness/hopelessness during the previous 12 months. Our results are also consistent with longitudinal research that found persistent food insecurity in childhood to be associated with internalizing and externalizing problems in adolescence, along with other psychosocial problems such as cannabis use, peer bullying, and school dropout, after differences in household income were controlled (Paquin et al., 2021; Slopen et al., 2010). Moreover, research in Canada on childhood hunger—a severe and less prevalent manifestation of food insecurity—found similar associations with depression and suicidal ideation in adolescence and early adulthood after controlling for household income (McIntyre et al., 2013, 2017).

This study addresses methodological weaknesses of previous investigations and found youth in more food-insecure households, irrespective of their household income, are more likely to experience a range of suicidal behaviours (Men et al., 2021). While we could not examine the causal mechanisms that underlie these associations given the study’s cross-sectional design, other research has identified physical and psychosocial pathways. First, food insecurity limits access to a variety of nutritious food options (Kirkpatrick & Tarasuk, 2008). Nutritional deficiencies in iron and calcium affect brain development and mental health through gut microbiota and other mechanisms (O’Neil et al., 2014; Owen & Corfe, 2017). Second, longitudinal evidence shows that food insecurity in childhood (or hunger, a severe manifestation of food insecurity) predicts poor mental health and other risk factors for youth suicide, including parental depression and domestic violence (Kirkpatrick et al., 2010; Melchior et al., 2009). Food insecurity may therefore be a proxy of unmeasured risk factors in the family. Third, food insecurity is a constant, powerful reminder of social disadvantage. The inability to afford food, having to cut or skip meals, and stigma associated with accepting donated foods are stressful experiences that contribute to mood disorders and suicidal behaviours through hypothalamic-pituitary-adrenocortical (HPA) pathways (Firth et al., 2020; Hamelin et al., 2002).

### Strengths and limitations

Strengths of the study include a large representative sample of youth, a well-validated multi-item scale of household food insecurity, and youth assessments of suicidal behaviours. The range of behaviours investigated is consistent with contemporary theoretical models of suicide. Interpersonal theory (Van Orden et al., 2010), three-step theory (Klonsky & May, 2015), and the integrated motivational-volitional model (O’Connor & Kirtley, 2018) all describe an ‘ideation-to-action’ framework which argues that the social determinants of suicidal thoughts or planning may differ from the determinants of a suicide attempt. Further epidemiological research is needed that recognizes this continuum of suicidal behaviour and the importance of youth suicide research to policy actions. Another strength was independent reports on food insecurity, household income, and suicidal behaviour, which limited the potential for shared method bias to inflate or diminish the associations found. Other surveys of youth health tend to rely on single informants and use rudimentary assessments of food insecurity (e.g. youth reports of hunger) or mental health (e.g. parent reports of youth symptoms), which is problematic given that parents and youth show low agreement in these domains (Jones et al., 2019; Landry et al., 2019). Their low agreement in food insecurity assessments may be attributed to parents’ shielding behaviours, in which they reduce their food intake and take other measures to prioritize their children’s nutritional needs and mask their worries about food (Coleman-Jensen et al., 2013; Hevesi et al., 2024). Conversely, most parents are unaware or deny the presence of suicidal behaviours in youth (Jones et al., 2019). Therefore, the multi-informant approach used in CHSCY was ideal for investigating these associations.

Limitations of the study should be noted as well. The exclusion of Indigenous communities, foster homes, and institutional settings from CHSCY limits the generalizability of these findings. The cross-sectional design precludes causal interpretations and analysis of transactional effects between variables. It should also be recognized that other adverse childhood experiences (often less easily measured) may have preceded food insecurity and partially mediated its associations with later distress and suicidal behaviour (Gundersen & Ziliak, 2014). Also, the single-item measures of suicidal behaviours used in CHSCY are prone to misclassification errors (Millner et al., 2015). More in-depth assessments, even with a subsample of youth, could have been useful for estimating and possibly correcting for those biases. Finally, although post-tax income would have better controlled socioeconomic differences in the sample, we had access to only pre-tax household income.

## Conclusion

Youth from food-insecure households are more likely to experience suicidal behaviours compared to their food-secure counterparts. While evidence gaps remain regarding early-life contextual factors that account for this association, there is an urgent need for coordinated policy actions that support at-risk youth through the reduction and elimination of food insecurity.

## Contributions to knowledge

What does this study add to the existing literature?

- Household food insecurity is associated with emotional and behavioural problems in children and youth.
- These associations are not fully explained by household income poverty and may involve the physical and mental health consequences of malnutrition and psychological stress.
- Its specific links to youth suicidal behaviours have not been examined in the Canadian context due to scarce data on suicide and inadequate controls for household income.
- This study examines the association of household food insecurity and self-reported suicidal behaviours in a nationally representative sample of Canadian youth.

What are the key implications for public health interventions, practice, or policy?

- Household food insecurity is negatively associated with youth well-being and mental health.
- Rising food insecurity in Canada and its robust association with suicidal behaviours highlight an urgent need for coordinated policy actions that improve access to foods and offer mental health supports to at-risk youth.

## Supplementary Information

Below is the link to the electronic supplementary material.

Supplementary file1 (DOCX 33 KB)

## Data Availability

Microdata from the CHSCY may be requested from Canadian Research Data Centre Network. The data used in this study are used under license for the current study by Statistics Canada and so are not publicly available.

## Code availability

The full dataset creation plan and analytic code are available from the authors upon request, understanding that the computer programs may rely upon coding templates or macros that are unique to CHSCY and are therefore either inaccessible or may require modification.
